# Synthesis of PDMS-μ-PCL Miktoarm Star Copolymers by Combinations (*Є*) of Styrenics-Assisted Atom Transfer Radical Coupling and Ring-Opening Polymerization and Study of the Self-Assembled Nanostructures

**DOI:** 10.3390/nano13162355

**Published:** 2023-08-17

**Authors:** Yi-Shen Huang, Dula Daksa Ejeta, Kun-Yi (Andrew) Lin, Shiao-Wei Kuo, Tongsai Jamnongkan, Chih-Feng Huang

**Affiliations:** 1 Department of Chemical Engineering, i-Center for Advanced Science and Technology (iCAST), National Chung Hsing University, Taichung 40227, Taiwan; yishen617@gmail.com (Y.-S.H.); duladaksa@gmail.com (D.D.E.); 2 Department of Environmental Engineering, i-Center for Advanced Science and Technology (iCAST), National Chung Hsing University, Taichung 40227, Taiwan; linky@nchu.edu.tw; 3 Department of Materials and Optoelectronic Science, Center for Nanoscience and Nanotechnology, National Sun Yat-Sen University, Kaohsiung 80424, Taiwan; kuosw@faculty.nsysu.edu.tw; 4 Department of Fundamental Science and Physical Education, Faculty of Science at Sriracha, Kasetsart University, Chonburi 20230, Thailand

**Keywords:** polydimethylsiloxane, poly(ε-caprolactone), miktoarm star copolymers, styrenics-assisted atom transfer radical coupling, combination

## Abstract

Due to their diverse and unique physical properties, miktoarm star copolymers (μ-SCPs) have garnered significant attention. In our study, we employed α-monobomoisobutyryl-terminated polydimethylsiloxane (PDMS-Br) to carry out styrenics-assisted atom transfer radical coupling (SA ATRC) in the presence of 4-vinylbenzyl alcohol (VBA) at 0 °C. By achieving high coupling efficiency (χ_c_ = 0.95), we obtained mid-chain functionalized PDMS-VBA_m_-PDMS polymers with benzylic alcohols. Interestingly, matrix-assisted laser desorption/ionization time of flight mass spectrometry (MALDI-TOF MS) analysis revealed the insertion of only two VBA coupling agents (m = 2). Subsequently, the PDMS-VBA_2_-PDMS products underwent mid-chain extensions using ε-caprolactone (ε-CL) through ring-opening polymerization (ROP) with an efficient organo-catalyst at 40 °C, resulting in the synthesis of novel (PDMS)_2_-μ-(PCL)_2_ μ-SCPs. Eventually, novel (PDMS)_2_-μ-(PCL)_2_ μ-SCPs were obtained. The obtained PDMS-μ-PCL μ-SCPs were further subjected to examination of their solid-state self-assembly through small-angle X-ray scattering (SAXS) experiments. Notably, various nanostructures, including lamellae and hexagonally packed cylinders, were observed with a periodic size of approximately 15 nm. As a result, we successfully developed a simple and effective reaction combination (*Є*) strategy (i.e., SA ATRC-*Є*-ROP) for the synthesis of well-defined PDMS-μ-PCL μ-SCPs. This approach may open up new possibilities for fabricating nanostructures from siloxane-based materials.

## 1. Introduction

Star copolymers (SCPs) are non-linear macromolecules with more than two linear polymer chains (arms) with a central core [1,2]. Among SCPs, homogeneous arms (e.g., homopolymer, random, or block copolymer segments) and heterogeneous arms (i.e., miktoarm SCPs (μ-SCPs)) are the two major classes of SCPs [3,4]. The former consists of arms with identical chemical structures, chain ends, and comparable compositions and molecular weights (MWs). The latter encompasses dissimilar arms with varying MWs, compositions, and chain-end functionalities linked at a central junction [5]. Their superior features of high functional group density and low solution viscosity, arising from a spatially defined and congested 3D globular structure, generally do not exhibit in typical linear copolymers [6].

Moreover, μ-SCPs have recently attracted significant attention due to their diversity, exhibiting unique physical properties. Originating from the architecture effects [5], the variability of compositions and functionality within μ-SCPs also grants access to a captivating range of novel properties, including distinct microdomain morphologies, the capacity to generate stable unimolecular micelles, and the formation of exceptional supramolecular assemblies. These unprecedented properties of μ-SCPs enable them in various potential applications, including thermoplastic elastomers, nanostructured thin films, solid-state electrolytes, gene and drug delivery systems, and stimuli-responsive materials [5,7,8,9]. Proper polymer designs with well-defined MWs, low molecular weight polydispersity (PDI), and controllable architectures thus play an essential role in establishing physical property relationships. This is vital for polymer chemists to achieve one of the most important goals of designing polymers with predetermined structures and manipulating materials with desired properties [10,11].

Based on the designing strategies, μ-SCPs can be synthesized via the core-first approach, coupled with the grafting-from or arm-first methods associated with the grafting onto/through approach. Through combinations (symbolized as “*Є*”) of two or more controlled/“living” polymerization (CLP) techniques, we can achieve control of the arm compositions, MWs, PDIs, and functionalities [6]. The CLP techniques include living anionic/cationic polymerization (LAP/LCP), reversible-deactivation radical polymerizations (RDRPs), ring-opening (metathesis) polymerization (ROP), catalyst transfer polymerization (CTP), chain-growth condensation polymerization (CGCP), etc. Using the core-first approach, μ-SCPs can be directly synthesized by initiating arm growth from a multifunctional initiator with heterogeneous sites through sequential initiation systems. Using the “core-first” approach, we might face the arduous synthesis of a multifunctional core and the later orthogonal initiating functionalities [12]. In addition, the synthesis of μ-SCPs with high MWs could be bargained by the occurrence of unsought terminations (e.g., intra-arms or inter-arms coupling reactions) during polymerization, leading to undesired molecular structures [13]. The arm-first approaches are more diverse. Several approaches can be utilized: (i) A conventional method is that heterogeneous polymer chains with a reactive end can be coupled with efficient organic reactions to conduct a grafting-onto strategy among the different types of polymer chain ends and the multifunctional core. (ii) A late-developed method is that macromonomer (MM) precursors can be first synthesized by CLPs or modified by a chain end of condensation-type polymers to possess a vinyl group. Subsequently, an appropriate amount of chain-end cross-linking via a CLP initiator can lead to μ-SCPs. (iii) Vice versa, macroinitiator (MI) precursors can be synthesized by CLPs or modified by the chain ends of condensation-type polymers to possess an initiating site. Subsequently, chain-end cross-linking through the CLP mechanism in the presence of divinyl compounds can also result in μ-SCPs [4,5]. (iv) A recently developed and innovative “in–out” strategy method was demonstrated. In this method, polymer arms were created, and a core was convergently formed through in-situ additions of divinyl crosslinking agents. Within the core of the SCP, dormant initiating sites were preserved. Subsequently, this star copolymer was utilized as a multifunctional MI to initiate CLP of another monomer, resulting in the production of μ-SCPs. 

Common drawbacks of the conventional arm-first approach are lack of chain mobility and steric hindrance in high MWs, leading to slow and inefficient reactions. Thus, it is usually employed for the syntheses of μ-SCPs with low MWs or fewer arms [13,14,15]. Conversely, the two recently developed arm-first methods offer a highly convenient and efficient means of preparing μ-SCPs. This is achieved by separately synthesizing functional arms with distinct compositions and crosslinking them together, leading to high molecular weight and a substantial number of arms (>100) [13,16]. It allows us to control the number of arms, which is dictated by parameters including amount of crosslinker, small amounts of monomers, timing of crosslinker addition, MW of arms, ratio of MMs/initiator, ratio of MIs/crosslinker, functionality, and composition of the polymeric arms [15,17,18].

In the previous literature, LAP has been extensively and successfully employed to synthesize various types of μ-SCPs since its pioneer position of CLPs [19,20,21,22,23,24,25,26,27,28,29]. It is still a challenge to extend the arm numbers beyond six in all cases. In recent decades, RDRP (e.g., atom transfer radical polymerization (ATRP), reversible addition–fragmentation chain-transfer (RAFT) polymerization, and nitroxide-mediated polymerization (NMP)) and ROP methods started a revolution owing to their orthogonal reactivity, gentle reaction conditions, and expansive monomer range in comparison to LAP [13,14,30,31,32,33,34,35,36,37]. Some reports on synthesizing polyester-based μ-SCPs by combining other chemistry and contemporary polymerization techniques and ROP are available. However, only a few works have reported using the arm-first route with the facile based on efficient radical coupling reactions, such as styrenics-assisted atom transfer radical coupling (SA ATRC) [5,16,38] and photoirradiation organotellurium-mediated radical coupling (*hv*TERC) [39,40] to synthesize polyester-based μ-SCPs. 

Several kinds of literature illustrate that SA ATRC is a unique method of introducing polymers with mid-chain functionality at a low temperature with high coupling efficiency (χ_c_) [13,31]. For example, Huang and co-workers progressively developed various A_n_B_m_-type μ-SCPs by using the arm-first approach via combinations of three techniques (e.g., ROP-*Є*-SA ATRC-*Є*-ATRP or ROP-*Є*-SA ATRC-*Є*-CGCP) [14,36]. In another case, the CGCP was first applied for the synthesis of well-defined poly(*N*-octyl benzamide) (PBA) and converted the chain end to an effective initiating site of 2-bromoisobutyryl (i.e., PBA-Br: *M*_n_ = 3000 and PDI = 1.1). A high coupling efficiency (>0.95) and doubling of the MW with low PDI (*M*_n_ = 5770 and PDI = 1.13) were obtained through the SA ATRC of PBA-Br with 4-vinylbenzyl alcohol (VBA) coupling agent. Through the combined strategy of CGCP-*Є*-SA ATRC-*Є*-ROP (i.e., final ROP mid-chain extension of PBA-VBA_m_-PBA MI with ε-caprolactone (ε-CL)), a novel well-defined (PBA)_2_-μ-(PCL)_4_ μ-SCP (*M*_n,NMR_ = ca. 13,400 and PDI = 1.17) was successfully obtained [41].

**Scheme 1:**
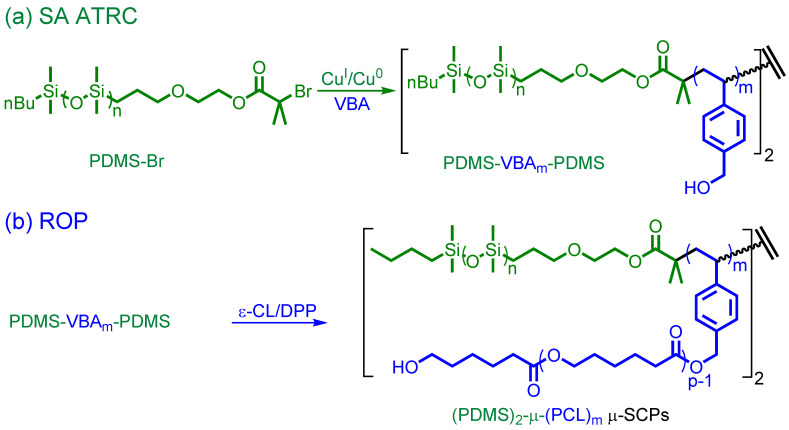
Reaction routes for the synthesis of μ-SCPs: (**a**) SA ATRC at 0 °C and (**b**) ROP at 40 °C.

Herein, we develop a simple and effective SA ATRC-*Є*-ROP strategy for synthesizing well-defined PDMS-μ-PCL (PDMS: polydimethylsiloxane) μ-SCPs. As shown in Scheme 1, the chain-end modification and SA ATRC of the PDMS-based precursors were conducted and examined by gel permeation chromatography (GPC) and matrix-assisted laser desorption/ionization time of flight mass spectrometry (MALDI-TOF MS). We employed a styrenic monomer, VBA, as a coupling agent to synthesize PDMS-VBA_m_-PDMS MI. An organo-catalyst catalyzed the ROP mid-chain extension of PDMS-VBA_m_-PDMS MI with ε-CL to afford well-defined μ-SCPs. Eventually, the self-assembly behaviors and the microstructures in the obtained solid-state PDMS-μ-PCL μ-SCPs were investigated by small-angle X-ray scattering (SAXS). 

## 2. Experimental Section

### 2.1. Materials

Triethylamine (TEA, 99.5%), 2-bromoisobutyryl bromide (BiB, 97%), copper bromide (CuBr, 99%), diphenyl phosphate (DPP, 97%), copper wire (Cu wire (diameter = 1.0 mm), 99.9%), hydrochloric acid (37%), formic acid (88%), magnesium sulfate anhydrous (MgSO_4_, 99%), dichloromethane (DCM, 99.9%), dimethyl sulfoxide (DMSO, 99%), diethyl ether (99%), methanol (MeOH, 99%), and tetrahydrofuran (THF, 99%) were procured from Sigma-Aldrich (St. Louis, MO, USA) and were used without purification. Monohydroxyl-terminated polydimethylsiloxane (PDMS-OH: *M*_n_ = 1250, PDI = 1.17) was purchased from Gelest. ε-Caprolactone (ε-CL, 99%) and toluene (99%) were dried by distillation with sodium hydride and sodium prior to use. According to the previous literature, we prepared 4-vinylbenzyl alcohol (VBA) [42,43,44,45] and tris(2-(dimethylamino)ethyl)amine (Me_6_TREN) [46,47,48] (Scheme S1a,b, respectively (see the ESI)). Their characterization of ^1^H NMR spectra is shown in Figure S1 (see the ESI).

### 2.2. Characterization

The progress of monomer conversion was tracked using a Hewlett Packard 5890 series II gas chromatograph (GC) featuring an FID detector and a CNW CD-5 column (30 m), with toluene employed as an internal standard. Gel permeation chromatography (GPC) was carried out at 40 °C in THF (flow rate: 1 mL/min) using a Waters 515 pump, a Waters 410 differential refractometer, a Waters 486 absorbance detector, and two PSS SDV columns (Linear S and 100 Å pore size) to determine *M*_n_, *M*_w_ and *M*_w_/*M*_n_ (i.e., PDI). Monodisperse polystyrene (PSt) standards were used for calibrations. Recycling preparative gel permeation chromatography (rGPC) was carried out at ambient temperature in THF (flow rate: 10 mL/min) using a LaboACE LC-5060 equipped with a P-LA60 pump, a RI-700 LA differential refractometer, and JAIGEL-2.5HR columns (exclusion limit 20,000). Polymer solutions (10 mg/mL) were purified to obtain low PDI samples. FT-IR spectra were captured using a Nicolet Avatar 320 FT-IR spectrometer operating at a resolution of 4 cm^−1^, with 24 scans. The samples were dissolved in THF and applied onto a KBr plate. Proton nuclear magnetic resonance (^1^H NMR) spectra were acquired using a Varian 400 NMR and calibrated against an internal standard of CDCl_3_ (δ = 7.26 ppm). Matrix-assisted laser desorption/ionization time-of-flight mass spectrometry (MALDI-TOF MS) was recorded on a Bruker Autoflex III spectrometer plus in the reflectron ion mode. Trans-2-[3-(4-*tert*-butylphenyl)-2-methyl-2-propenylidene] malononitrile (DCTB) and sodium chloride (NaCl) were, respectively, used as the matrix and ionizing agent for the MALDI-TOF mass measurements. The dried samples were placed in an N_2_-purged chamber to maintain environmental dryness. Small-angle X-ray scattering (SAXS) analysis was conducted at the Endstation BL23A1 National Synchrotron Radiation Research Center (NSRRC) in Hsinchu, Taiwan. The X-ray source operated at 15 kV, and the sample-to-detector distance was 3 m. The scattering profile, obtained using a Pilatus-1MF detector, was plotted in terms of scattering intensity (I) as a function of the scattering vector magnitude [q = (4π/λ) sin (θ/2)]. The d-spacing was determined from the first-order scattering peak (q*) using the formula d = 2π/q*.

### 2.3. Synthesis of PDMS-Br Macroinitiator (MI)

An acid scavenger TEA (1 mL, 9.9 mmol), PDMS-OH (5.5 g, 5 mmol), and DCM (50 mL) were mixed in a reaction flask. BiB (2.3 mL, 9.9 mmol) was added dropwise into the solution. The reaction mixture was stirred at room temperature for 24 h. The reaction mixture was diluted with 1 M HCl solution (100 mL) and extracted with DCM 100 mL 3 times. The collected organic phase was washed with deionized water, dried with MgSO_4,_ and concentrated to obtain PDMS-Br precursor (*M*_n,NMR_ = 1280, *M*_n,GPC_ = 1280, PDI = 1.14, yield 80%). ^1^H NMR (400 MHz, CDCl_3_, δ = ppm): 4.32 (t, -C*H*_2_OCO-, 2H), 3.67 (t, -C*H*_2_CH_2_OCO-, 2H), 3.45 (t, -CH_2_CH_2_C*H*_2_O-, 2H), 1.94 (s, -OCOC(C*H*_3_)_2_Br, 6H), 1.60 (m, -CH_2_C*H*_2_CH_2_O-, 4H), 1.25–1.37 (m, CH_3_C*H*_2_C*H*_2_CH_2_-, 4H), 0.88 (t, C*H*_3_CH_2_CH_2_CH_2_-, 3H), 0.53 (m, -Si(CH_3_)_2_C*H*_2_-, 4H), 0.1 (br, -(C*H*_3_)_2_ of PDMS, 6H).

### 2.4. Synthesis of PDMS-VBA_m_-PDMS through Styrenics-Assisted Atom Transfer Radical Coupling (SA ATRC)

In an example of the SA ATRC reaction, CuBr (0.43 g, 3 mmol), toluene (100 mL), and a stirring bar wrapped with fresh copper wire (5 cm) were added to a septum-sealed Schlenk flask. The mixture was subsequently deoxygenated by nitrogen purging treatment for 60 min. A mixture of VBA (3.8 mL, 30 mmol), PDMS-Br (3.8 g, 3 mmol with *M*_n,0_ = 1280, PDI = 1.14), and Me_6_TREN (2 mL, 7.5 mmol) was then injected into the flask after treating in the nitrogen atmosphere and the reaction was cooled to 0 °C. Samples were taken periodically under a nitrogen blanket and passed through a short column of neutral alumina to remove dissolved copper salts before the analysis of GPC. The reaction was exposed to an ambient atmosphere and the crude product was diluted with THF and passed through a neutral alumina column. The solution was concentrated under a vacuum to remove the residual solvent, monomer, and ligand. The collected polymer was vacuum-dried to afford PDMS-VBA_m_-PDMS (*M*_n_ = 2440, PDI = 1.31, yield 25%; coupling efficiency (*x*_c_) = 0.95 where *x*_c_ = 2 × (1 − *M*_n,0_/*M*_n_)).

### 2.5. Chain Extension of PDMS-VBA_m_-PDMS with ε-CL through ROP

An oven-dried Schlenk flask and magnetic stirrer bar with DPP (50 mg, 0.2 mmol) and PDMS-VBA_m_-PDMS (0.5 g, 0.2 mmol) and toluene (10 mL) were charged. The flask was sealed and deoxygenated using freeze–pump–thaw for three cycles and refilled with nitrogen. ε-CL (1.1 mL, 9.8 mmol) was injected into the solution ([ε-CL]_0_ = 0.8 M), and the polymerization was allowed to commence at 40 °C. An initial sample was taken via a syringe to trace the chain extension through ROP at ambient temperature. The reaction was stopped by adding an acidic Amberlyst^®^ A21 and diluted with THF. The ion-exchange resin was removed by filtration and the clear solution was precipitated in MeOH. Using similar procedures, two crude PDMS-μ-PCL were collected and dried under vacuum (i.e., *M*_n,GPC_ = 4300, PDI = 1.72, yield 75%; *M*_n,GPC_ = 7990, PDI = 1.52, yield 73%). These samples were further purified by rGPC to remove the impurities and well-defined PDMS-μ-PCL samples can be acquired (i.e., μ-SCP1: *M*_n,GPC_ = 7140, PDI = 1.24 and μ-SCP2: *M*_n,GPC_ = 11,230, PDI = 1.20).

## 3. Results and Discussion

**Figure 1:**
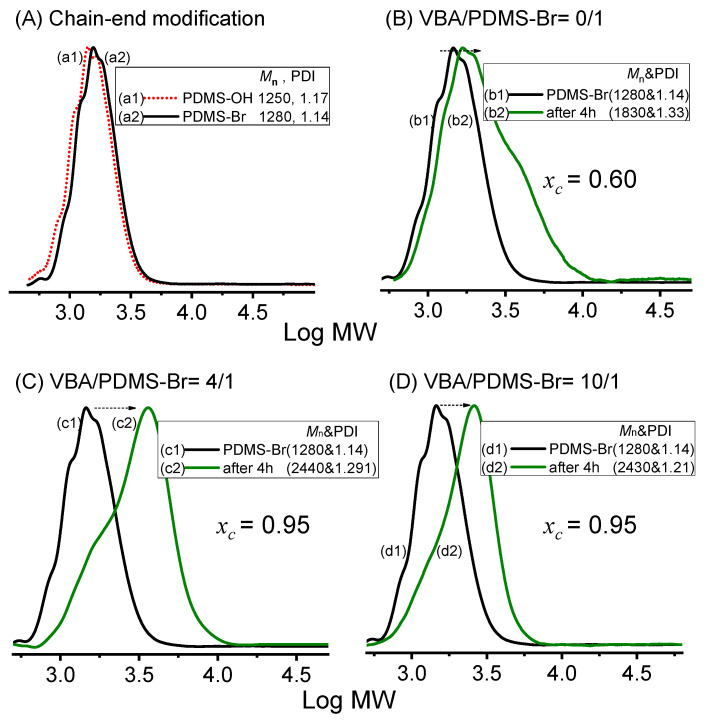
(**A**) GPC traces for the PDMS chain-end modification. Monitoring the coupling efficiency (*x_c_*) by GPC traces with various VBA/PDMS-Br ratios: (**B**) 0/1, (**C**) 4/1, and (**D**) 10/1 (VBA/PDMS-Br/CuBr/Me_6_TREN = x/1/1/2.5 at 0 °C with 0.05 cm copper wire/mL toluene; [PDMS-Br]_0_ = 0.03 M in toluene; *x_c_* = 2 × (1 − *M*_n,0_/*M*_n_)).

Figure 1A demonstrates the GPC traces of PDMS-OH and PDMS-Br. Before conducting effective SA ATRC [49,50,51,52], the PDMS-Br precursor was synthesized through the acylation of the PDMS-OH with 2-bromoisobutyryl bromide (Scheme S1c (see the ESI)) [47]. The GPC traces of the corresponding PDMS-OH and PDMS-Br products (i.e., curves a1 and a2 in Figure 1A) showed only a minor shift and remained similar in molecular weight (MW) characteristics. After acylation of PDMS-OH, the MW slightly shifted from 1250 to 1280 with low PDI values (PDI < 1.17). Characterized by IR spectroscopy, Figure S2a (see the ESI) displays the bending of the Si-CH_3_ bond, Si-O, Si-CH_3_ bond, and the stretching of the C-H bond (790, 1010, 1260, and 2960 cm^−1^, respectively). As shown in Figure S2b (see the ESI), the stretching of C=O (1740 cm^−1^) was additionally observed, revealing the successful acylation of the PDMS-OH. As shown in Figure 1B–D, various ratios of VBA/PDMS-Br were then conducted to examine the efficacy of SA ATRCs (VBA/PDMS-Br/CuBr/Me_6_TREN = x/1/1/2.5 at 0 °C with 0.05 cm Cu wire/mL toluene (x = 0, 1, 4, and 10)). Without VBA (Figure 1B), the *M*_n_ increased to approximately 1830 with a low PDI value (1.33) after SA ATRC of PDMS-Br. The coupling efficiency (*x*_c_) can be estimated to be ca. 0.6 (where *x*_c_ = 2 × (1 − *M*_n,0_/*M*_n_), *M*_n,0_: the initial number average MW, *M*_n_: the number average MW after coupling). For SA ATRCs with excess amounts of VBA (Figure 1C,D), the *M*_n_s increased to approximately double the *M*_n,0_ (ca. 2450) with low PDI values (<1.30). High *x*_c_ values of approximately 0.95 were acquired in both cases. With a high ratio of VBA/PDMS-Br = 10, the result stayed at the coupling reaction instead of the chain extension of the PDMS-Br with VBA. This is ascribed to the low-temperature condition for SA ATRC.

**Figure 2:**
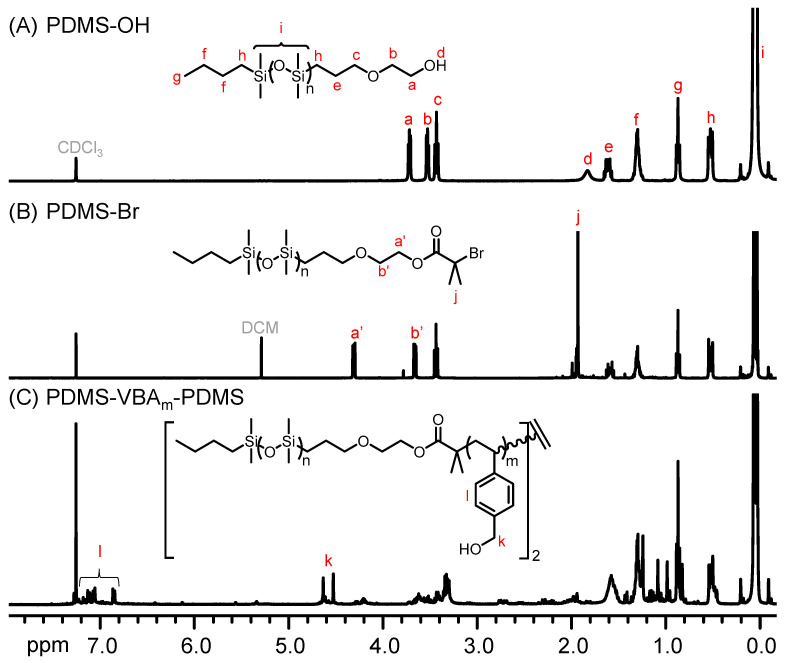
^1^H NMR spectra (400 MHz, CDCl_3_) of (**A**) PDMS-OH, (**B**) PDMS-Br, and (**C**) PDMS-VBA_m_-PDMS.

Figure 2 displays ^1^H NMR spectroscopy analysis of the PDMS-based polymers and their assigned relevant characteristic peaks. Compared the spectra of 2A and 2B, quantitative chain end modification from PDMS-OH to PDMS-Br can be confirmed (i.e., disappearance of peaks a (~3.65 ppm (t, -CH_2_C*H*_2_OH, 2H)) and b (~3.45 ppm (t, -C*H*_2_CH_2_OH, 2H)) and appearance of peaks a’ (~4.32 ppm (t, -C*H*_2_OCO-, 2H)), b’ (~3.67 ppm (t, -C*H*_2_CH_2_OCO-, 2H)), and j (~1.94 ppm (s, -OCOC(C*H*_3_)_2_Br, 6H))). After SA ATRC of PDMS-Br with VBA (Figure 2C), the disappearance of peak j and appearance of peaks k and l were observed (~4.6 and 6.8–7.2 ppm, respectively). The ^1^H NMR spectra implied the occurrence of the convergent reaction that inserted only a few VBA units.

**Figure 3:**
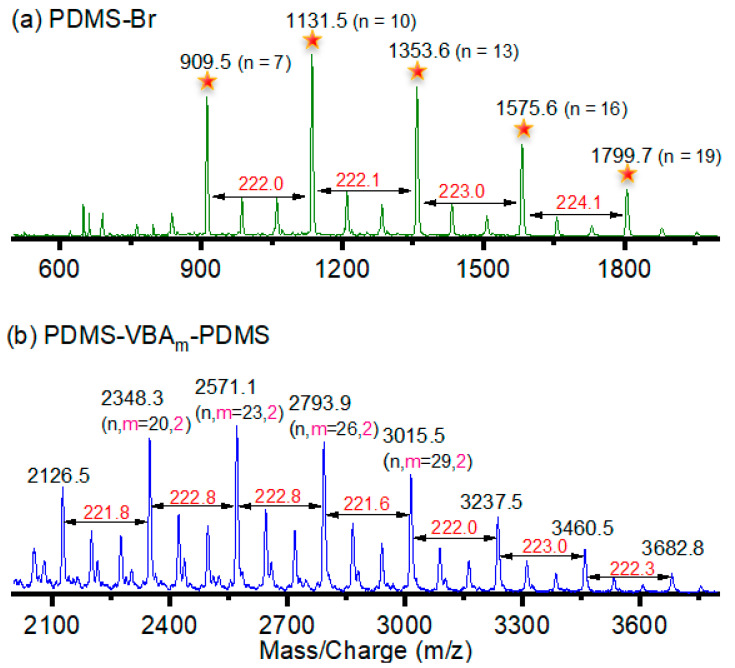
MALDI-TOF MS of (**a**) PDMS-Br and (**b**) PDMS-VBA_m_-PDMS polymers (n: DMS units, m: VBA units).

To further understand the structural details after SA ATRC, the PDMS-Br precursor and a well-coupled PDMS-VBA_m_-PDMS polymer were analyzed by MALDI-TOF MS using DCTB as the matrix and NaCl as the ionizing agent. Figure 3a displays the MALDI-TOF spectrum of PDMS-Br (*M*_n_ = 1280, PDI = 1.14). Interestingly, a series of major peaks (i.e., star marks) with a range of approximately 222 Da, corresponding to the hexamethyl trisiloxane (HMTS) molar mass, was observed. This should intrinsically originate from the ROP of the 6-member ring of HMTS for PDMS preparations. Taking an example of the peak at *m*/*z* = 1131.5, the PDMS-Br with rational functionalities can be deduced (i.e., *m*/*z* (cal.) = MW_head_ (115.27) + n × MW_DMS unit_ (10 × 74.15) + MW_end_ (252.13) + MW_Na_+ (22.99) = 1131.89), indicating alternative evidence of the chain end modification through robust MALDI-TOF MS analysis. Figure 3b shows the MALDI-TOF spectrum of PDMS-VBA_m_-PDMS (*M*_n_ = 2430 g/mol, PDI = 1.21), which also displayed a series of major peaks corresponding to the HMTS repeating unit (ca. 222 Da). Taking an example of the peak at *m*/*z* = 2348.3, structural details of the coupling product can be estimated (i.e., *m*/*z* (cal.) = MW_chain-ends_ (2 × 115.27) + n × MW_DMS unit_ (20 × 74.15) + m × MW_VBA_ (2 × 134.18) + MW_mid-linkages_ (2 × 172.22) + MW_Na_+ (22.99) = 2349.3). These results importantly and surprisingly revealed that high *x*_c_ of SA ATRC can be attained even at 0 °C and only two VBA coupling agents (m = 2) were inserted. 

The scenario can be rationalized based on kinetic details to discuss such interesting results. For simplicity, the rate constants of activation (*k*_act_), deactivation (*k*_dea_), and termination (*k*_t_) of PDMS-Br with the bromoisobutyryl group are classified to those of the similar structures of ethyl 2-bromoisobutyrate (EBiB) and methyl methacrylate (MMA) (i.e., *k*_act,EBiB_, *k*_dea,EBiB_, and *k*_t,MMA_, respectively). For the role of VBA, the rate constants were amended from 1-phenylethylbromide (PEBr) and styrene (St) (i.e., *k*_act,PEBr_, *k*_dea,PEBr_, *k*_p_, and *k*_t,St_). Table 1 summarizes the relevant reaction rate constants. In the absence of VBA, disproportionation termination between PDMS• methacrylic macroradicals was dominated [53,54], leading to the formation of PDMS-= and PDMS-H products. Once VBA was added, disproportionation termination of the PDMS• methacrylic macroradical was significantly suppressed and guided the overall reaction to conduct radical–radical coupling based on the following kinetic balances: (i) A high *k*_dea,EBiB_ (= ca. 5.1 × 10^5^ M^−1^ s^−1^) [55,56,57] that can conduct efficient deactivation reaction and thus keep the PDMS-Br chain-end integrity; (ii) A rapid cross-reaction between PDMS• and VBA (*k*_cr_ = ca. 2.5 × 10^3^ M^−1^ s^−1^) [58] to significantly transform the nature of the methacrylic macroradical to styrenic macroradical; (iii) The propagation rate constant of VBA is maintained at a significantly low value of approximately 40 M^−1^ s^−1^ at 0 °C. This critical control guarantees minimal chain extension during the reaction, thereby minimizing the consecutive addition of VBA to the newly-formed PDMS-VBA• macroradical (i.e., resulting in nearly a monoadduct). The prompt coupling reaction can be effectively dominated (*k*_t,St_ = ca. 10^8^ M^−1^ s^−1^) due to the nature of the styrenic radical termination [54,59,60]. Rationally, the *k*_act,PEBr_ and *k*_dea,PEBr_ are relatively low, which would be insufficient to establish the activation–deactivation reactions of PDMS-VBA-Br. Thus, PDMS-VBA_2_-PDMS was mainly formed. 

**Figure 4:**
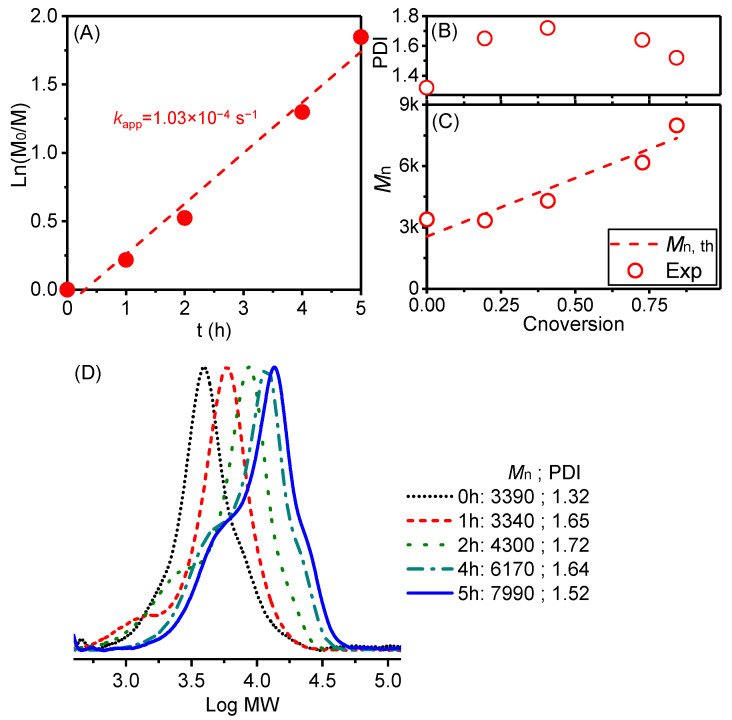
Chain extension of PDMS-VBA_2_-PDMS with ε-CL: (**A**) Ln vs. t, (**B**,**C**) PDI/*M*_n_ vs. Conversion, and (**D**) GPC traces for the ROP chain extension (ε-CL/PDMS-VBA_2_-PDMS/DPP = 50/1/1 at 40 °C; [ε-CL]_0_ = 0.8 M).

**Figure 5:**
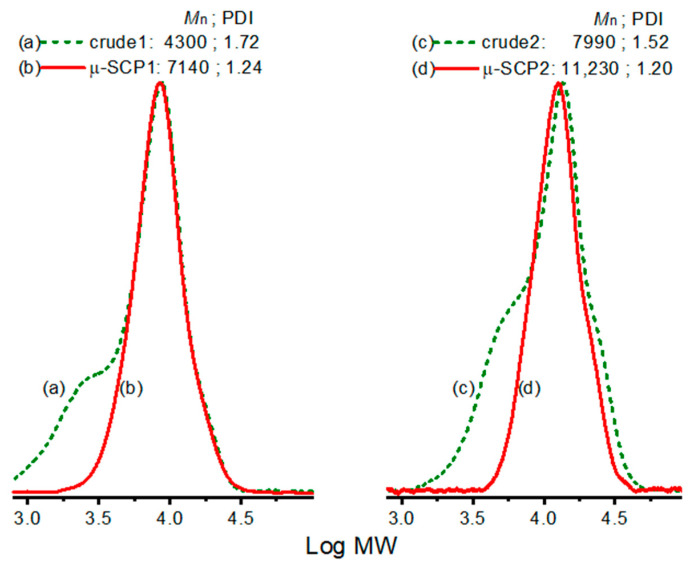
GPC traces for the (a, c) crudes and the corresponding rGPC-purified products of (b) μ-SCP1 and (d) μ-SCP2.

After achieving high efficiency of SA ATRC with VBA as a coupling agent, mid-chain functionalized polymers with benzylic alcohols were obtained. The PDMS-VBA_2_-PDMS MI with two hydroxyl groups allows us to perform chain extension with ε-CL through living-ROP (ε-CL/PDMS-VBA_2_-PDMS/DPP = 50/1/1 at 40 °C; [ε-CL]_0_ = 0.8 M). As shown in Figure 4A, the kinetics revealed a linear first-order plot with an apparent rate constant (*k*_app_) of approximately 1.03 × 10^−4^ s^−1^. Figure 4B showed moderate increases in PDIs and Figure 4C showed gradual increases in MWs and the *M*_n_s relating to conversion. As shown in Figure 4D, the GPC traces showed significant growth in MW. However, a shoulder was observed after 2 h, which might be attributed to the unignorable self-polymerization of ε-CL via the cationic catalyst. To obtain the well-defined PDMS-μ-PCL μ-SCPs with low PDI, we employed a recycling GPC (rGPC) to separate the undesired portions. As shown in Figure S3, the trace during the first cycle (elution time: 7.7–13 min) revealed poor separation due to insufficient elution time. The trace displayed sufficient separation time in the second cycle (elution time: 17.7–27 min). The desired PDMS-μ-PCL was collected within an elution time of 18–23 min. As shown in Figure 5, curves a and c with shoulders possessed relatively large PDIs (>1.5). After the rGPC purifications, curves b and d displayed low PDIs (<1.3) with mono-modal traces, accompanied by increased MWs, indicating the undesired products had been effectively purified. 

**Figure 6:**
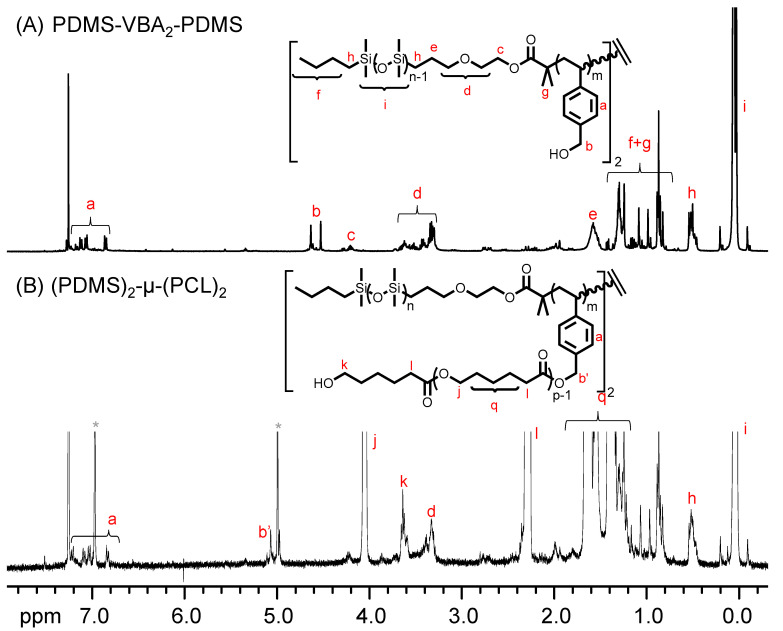
^1^H NMR spectra (400 MHz, CDCl_3_) of (**A**) PDMS-VBA_2_-PDMS and (**B**) (PDMS)_2_-μ-(PCL)_2_ (*: inhibitor peaks originating from THF solvent).

The obtained PDMS-μ-PCL μ-SCP was also subjected to structural elucidation using ^1^H NMR spectroscopy. Figure 6A displays the spectrum of PDMS-VBA_2_-PDMS MI, which contains benzylic alcohol (-Ph-C*H*_2_-OH) at peak b (4.4–4.7 ppm). In Figure 6B, the spectrum of PDMS-μ-PCL μ-SCP reveals the presence of characteristic signals from the PDMS backbone. Additionally, peaks b’, j, l, and q from benzyl ester and PCL backbone (4.9–5.2, 4.06, 2.30, 1.2–1.7 ppm, respectively) were observed, while peak b corresponding to the benzylic alcohol disappeared. Two samples were thus acquired and the volume fractions of PDMS in μ-SCP1 and μ-SCP2 were estimated as *f*^v^_PDMS_ = 0.46 and 0.32, respectively. The successful acquisition of μ-SCP1 (*M*_n,GPC_ = 7140, PDI = 1.24) and μ-SCP2 (*M*_n,GPC_ = 11,230, PDI = 1.20) was achieved using the rGPC instrument, and both samples exhibited well-defined structures (see again curves b and d in Figure 5).

**Figure 7:**
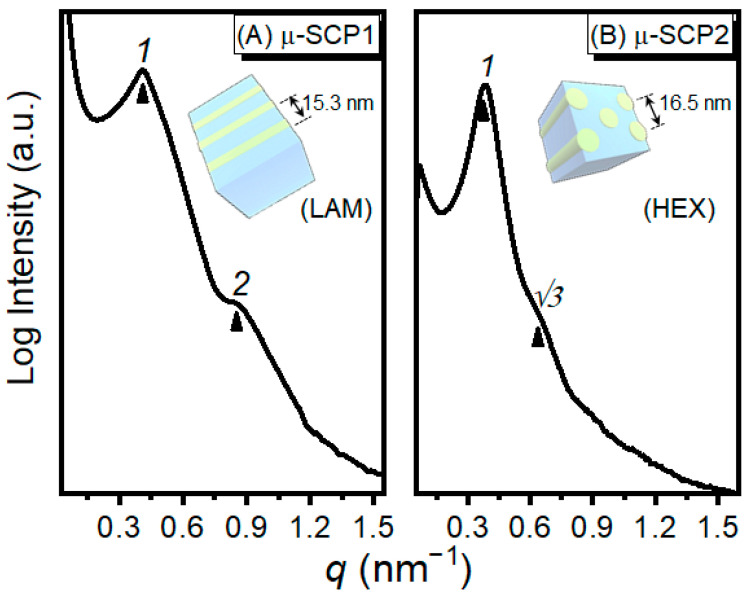
SAXS profiles of thermal annealed PDMS-μ-PCL of (**A**) μ-SCP1 (*M*_n,GPC_ = 7140, *f*^v^_PDMS_ = 0.46) and (**B**) μ-SCP2 (*M*_n,GPC_ = 11,230, *f*^v^_PDMS_ = 0.32) bulk film samples.

**Figure 8:**
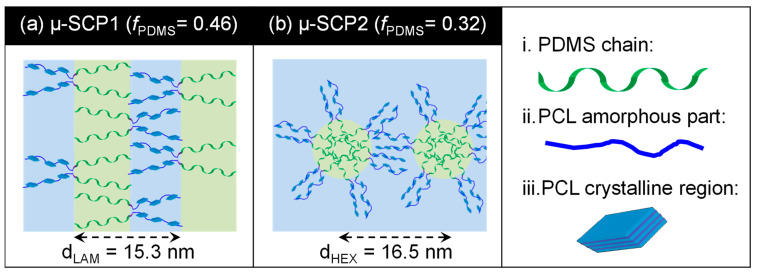
Proposed microstructures of the annealed μ-SCP bulk film samples referred to the SAXS analysis and estimated contour length.

The obtained μ-SCP1 and μ-SCP2 were further investigated for their solid-state self-assembly. Bulk film samples were individually prepared by solution-casting method (5 wt% μ-SCPs/THF solution) on Teflon plates. The specimens were dried under ambient temperature for 24 h and then thermal annealed at 60 °C under vacuum for 12 h. The obtained bulk film samples were measured using SAXS under ambient conditions to analyze their microstructure. As shown in Figure 7A, a lamellar (LAM) structure (i.e., q/q* peaks of 1:2) were obtained in the annealed sample of μ-SCP1 [(PDMS_13_)_2_-μ-(PCL_20_)_2_, *f*^v^_PDMS_ = 0.46]. The d-spacing of the nanostructures was approximately 15.3 nm. As shown in Figure 7B, a hexagonally packed cylinder (HEX) structure (i.e., q/q* peaks of 1:√3) having a periodic size of approximately 16.5 nm was detected in the case of the μ-SCP2 sample [(PDMS_13_)_2_-μ-(PCL_38_)_2_, *f*^v^_PDMS_ = 0.32]. It is worth noting that the “bottom-up” approaches to forming significantly ordered nanostructures by block copolymer (BCP) have a common strategy to use overall high molecular weight copolymers (i.e., high degrees of polymerization (*N*)) to conduct self-assembly behaviors. It is generally because most polymer–polymer interaction parameters (χ) are small. However, Kim et al. [62]. introduced an alternative strategy using a star block copolymer (SBCP) with 18-armed PMMA-*b*-PSt (i.e., (PMMA-*b*-PSt)_18_). They introduced a self-neutralization approach to achieve structured nanoarchitectures by increasing the “entropy penalty”. By innovatively controlling the polymer architecture in such a low χ system (ca. 0.04) [63], they beautifully fabricated oriented lamellar and cylindrical nanostructures ranging from approximately 50–150 nm on silicon wafers. Similarly, Ho et al. [64] combined two strategies to produce aligned structures (i.e., low entropy and large χ). By controlling the polymer architecture of (PSt_x_-*b*-PDMS_y_)_n_ SBCPs (arm numbers (n) = 1–4 and repeating units (x + y) < 20), they ensured a high value of χ (ca. 0.27) between the blocks. As a result, these SBCPs spontaneously aligned into the cylinder and lamellar structures of sub-20 nm feature size on silicon wafers. A similar two-strategies method was interestingly demonstrated by a unique system of maltoheptaose-based SBCPs [65,66], which showed sub-10 nm feature size. Our PDMS-μ-PCL system has the attributes of low *N*, large χ, and low entropy. Accordingly, such peculiarities can easily lead to performing self-assembly on a sub-nano scale. By minimizing the molecular spatial stability through MM2 molecular modeling method, contour lengths (L) of the μ-SCPs can be estimated. As shown in Figure S4 (see the ESI), L_μ-SCP1_ = 22.7 and L_μ-SCP2_ = 36.9 nm were acquired based on a set of end groups, PDMS_n_ segments, PCL_p_ segments, linkages, and VBA units. Figure 8 displays our proposed microstructure to indicate the roles and features of PDMS (i.e., only in the form of amorphous) and PCL (i.e., with the forms of amorphous and crystalline) chains. Consequently, the measured periodic dimensions of the μ-SCPs using SAXS are smaller than the estimated contour lengths. This phenomenon can be logically attributed to the strong crystallization integrity of the PCL chains, leading to a densely packed PCL-rich area, whether in a LAM or HEX structure. Our ongoing efforts involve examining the intrinsic properties and self-assembly tendencies of these μ-SCPs. More comprehensive insights and specific findings will be unveiled in an upcoming publication.

## 4. Conclusions

We employed a styrenic coupling agent, VBA, in the SA ATRC process to obtain PDMS-VBA_m_-PDMS products. By analyzing the outcomes through GPC, we achieved a high coupling efficiency (χ_c_ = 0.95) with a monomodal MW distribution. Intriguingly, MALDI-TOF MS analysis of the PDMS-VBA_m_-PDMS product confirmed the insertion of only two VBA units (m = 2). To extend chains, we subjected PDMS-VBA_2_-PDMS to mid-chain extensions with ε-CL through ROP, employing the organo-catalyst DPP. To remove any undesirable side products, such as linear PCL homopolymer, we employed a rGPC process. This allowed us to acquire well-defined μ-SCPs with low PDIs (<1.30). Further examination of the obtained PDMS-μ-PCLs through SAXS measurements revealed microphase separations, with LAM and HEX structures observed in bulk film samples of μ-SCP1 (*M*_n,GPC_ = 7140, *f*^v^_PDMS_ = 0.46) and μ-SCP2 (*M*_n,GPC_ = 11,230, *f*^v^_PDMS_ = 0.32), respectively. The achievement of sub-15 nm microstructures can be attributed to the attributes of low PDI (<1.3), low *N* of arms (PDMS_13_ and PCL_20_/PCL_38_), large χ, and low entropy in the μ-SCPs. In brief, our approach demonstrates high coupling efficiency and provides a means to introduce mid-chain functionality for conducting post-reactions. This convergent strategy can be extended to synthesize architecturally distinct and well-defined novel macromolecules. The characterization of structural details and diversities is currently underway. We will present the relevant data shortly. 

## Figures and Tables

**Table 1:** Reaction rate constants at 0 °C based on the relevant initiators (EBiB and PEBr) and monomers (MMA and St).

Rate Constant	Value (M^−1^ s^−1^)	Reference
*k*_act,EBiB_	13	[55,56,57]
*k*_dea,EBiB_	5.1 × 10^5^	[55,56,57]
*k*_t,MMA_	6.0 × 10^8^	[61]
*k*_cr_	2.5 × 10^3^	[58]
*k*_p_	42	[55,56,57]
*k*_t,St_	4.0 × 10^8^	[59]
*k*_act,PEBr_	0.8	[55,56,57]
*k*_dea,PEBr_	1.8 × 10^4^	[55,56,57]

*k*_act_: ATRP activation rate constant; *k*_dea_: ATRP deactivation rate constant. These rate constants were determined based on the conditions outlined in the literature (specifically, values for EBiB and PEBr with CuBr/Me_6_TREN in MeCN). *k*_cr_: Rate constant for cross-reaction from methacrylic radical to St (value calculated from the activation energy and frequency factor). *k*_p_: Propagation rate constant for styrenics; *k*_t_: Termination rate constant.

## Data Availability

Figure 7 is the data from the NSRRC.

## Supplementary Materials

The following supporting information can be downloaded at: https://www.mdpi.com/article/10.3390/nano13162355/s1, Scheme S1. Reaction steps for the syntheses of (a) VBA, (b) Me_6_TREN, and (c) PDMS-Br (BiB: 2-bromoisobutyryl bromide). (d) Cyclic monomer of hexamethyl trisiloxane. Figure S1. ^1^H NMR spectra (400 MHz, CDCl_3_) of (a) VBA and (b) Me_6_TREN. Figure S2. FT-IR spectra of (a) PDMS-OH and (b) PDMS-Br. Figure S3. A consecutive rGPC trace for purifying a PDMS-μ-PCL crude product [red region: collections of well-defined μ-SCPs (i.e., elution time = ca. 18–23 min)]. Figure S4. Spatial contour lengths of the relevant functionalities and monomer (estimated through MM2 molecular modeling) for L_μ-SCP1_ = 22.7 and L_μ-SCP2_ = 36.9 nm [estimation of the extended distance (L) using one set of PDMS end, PDMS_n_ segment, PCL_p_ segment, linkage, and VBA unit].
