# Supplementary material for: Synthesis of PDMS-μ-PCL Miktoarm Star Copolymers by Combinations (Є) of Styrenics-Assisted Atom Transfer Radical Coupling and Ring-Opening Polymerization and Study of the Self-Assembled Nanostructures
Source: Nanomaterials (Basel). 2023 Aug 17;13(16):2355. doi: 10.3390/nano13162355 (PMC10457737; doi:10.3390/nano13162355)
Supplement: Supplementary file 1 [file nanomaterials-13-02355-s001.zip › nanomaterials-2565571-supplementary.pdf]

Supporting Information for

Synthesis of PDMS-μ-PCL Miktoarm Star Copolymers by Combinations (*Ɛ*) of Styrenics-Assisted Atom Transfer Radical Coupling and Ring-Opening Polymerization and Study of the Self-Assembled Nanostructures

Yi-Shen Huang [1,†], Dula Daksa Ejeta [1,†], Kun-Yi (Andrew) Lin [2], Shiao-Wei Kuo [3], Tongsai Jamnongkan [4,*] and Chih-Feng Huang [1,*]

[1] Department of Chemical Engineering, i-Center for Advanced Science and Technology (iCAST), National Chung Hsing University, Taichung 40227, Taiwan; yishen617@gmail.com (Y.-S.H.); duladaksa@gmail.com (D.D.E.)

[2] Department of Environmental Engineering, i-Center for Advanced Science and Technology (iCAST), National Chung Hsing University, Taichung 40227, Taiwan; linky@nchu.edu.tw

[3] Department of Materials and Optoelectronic Science, Center for Nanoscience and Nanotechnology, National Sun Yat-Sen University, Kaohsiung 80424, Taiwan; kuosw@faculty.nsysu.edu.tw

[4] Department of Fundamental Science and Physical Education, Faculty of Science at Sriracha, Kasetsart University, Chonburi 20230, Thailand

* Correspondence: jamnongkan.t@ku.ac.th (T.J.); huangcf@dragon.nchu.edu.tw (C.-F.H.)

† These authors contributed equally to this work.

Captions.

Experimental Section.

Scheme S1. Reaction steps for the syntheses of (a) VBA, (b) Me_6TREN, and (c) PDMS-Br (BiB: 2-bromoisobutyryl bromide). (d) Cyclic monomer of hexamethyl trisiloxane.

Figure S1. ^1H NMR spectra (400 MHz, $CDCl_3$) of (a) VBA and (b) Me_6TREN.

Figure S2. FT-IR spectra of (a) PDMS-OH and (b) PDMS-Br.

Figure S3. A consecutive rGPC trace for purifying a PDMS-μ-PCL crude product [red region: collections of well-defined μ-SCPs (i.e., elution time = ca. 18–23 min)].

Figure S4. Spatial contour lengths of the relevant functionalities and monomer (estimated through MM2 molecular modeling) for $L_{\mu\text{-SCP1}}$ = 22.7 and $L_{\mu\text{-SCP2}}$ = 36.9 nm [estimation of the extended distance (L) using one set of PDMS end, $PDMS_n$ segment, PCL_p segment, linkage, and VBA unit].

Experimental Section.

Materials.

Formaldehyde aqueous solution (37%), sodium acetate (98%), sodium borohydride ($NaBH_4$, 98%), ethanol (EtOH, 99%), sodium chloride (NaCl, 99%), sodium hydroxide (NaOH, 97%), tris(2-aminoethyl)amine (TREN, 96%) 4-vinylbenzyl chloride (VBC, 90%), dichloromethane (DCM, 99.9%), dimethyl sulfoxide (DMSO, 99%), diethyl ether (99%), and tetrahydrofuran (THF, 99%) were purchased from Sigma-Aldrich and used without purification.

Synthesis of 4-vinylbenzyl alcohol (VBA).

As shown in Scheme S1a, a reaction flask containing VBC (18.5 mL, 131 mmol), sodium acetate (12.9 g, 157 mmol) and DMSO (60 mL) was immersed in a 45 °C water bath allowed stirring for 24 h. The reaction was quenched to room temperature, then deionized water (150 mL) was added. The dilute solution was extracted by ethyl ether. The collected organic phase was washed with brine, dried with $MgSO_4$ and concentrated to obtain VBAc crude product. A solution of sodium hydroxide (11.5 g, 288 mmol) and deionized water (46 mL) was mixed with VBAc and EtOH (50 mL) in a reaction flask. The reaction was conducted in an open system at 80 °C for 3 h. The solution was concentrated, followed by extraction with ethyl ether. The collected organic phase was washed with brine, dried with $MgSO_4$ and concentrated. The crude product was distilled at 90 °C under vacuum yield colorless oil (11.7 g, yield 66%). ^{1}H NMR (400 MHz, $CDCl_3$, δ = ppm): 7.30–7.41 (m, benzene ring, 4H), 6.68–6.75 (dd, -$CH_2C\mathit{H}$-, 1H), 5.73–5.78 (d, $C\mathit{H}_2$CH-, 1H), 5.24–5.27 (d, $C\mathit{H}_2$CH-, 1H), 4.65 (s, -$C\mathit{H}_2$OH, 2H).

Synthesis of tris[2-(dimethylamino)ethyl]amine (Me_6TREN).

As shown in Scheme S1b, TREN (6 mL, 40 mmol), 88% formic acid (38.2 g, 729 mmol) and MeOH (300 mL) were mixed in a reaction flask. A 37% formaldehyde solution was added dropwise into the flask, stirring at room temperature for 1 h. Sodium borohydride (11.1 g, 293 mmol) powder was slowly added to the stirred solution at 0 °C. The mixture was stirred at room temperature for an external 48 h and condensed under vacuum. A 3 M sodium hydroxide solution (250 mL) was added to the flask and extracted with DCM. The collected organic phase was washed with brine, dried with $MgSO_4$ and concentrated. The collected organic phase was washed with brine, dried with $MgSO_4$ and concentrated. The crude product was distilled at 100 °C under vacuum yield colorless oil (3.9 g, yield 42%). ^1H NMR (400 MHz, $CDCl_3$, δ = ppm): 2.58–2.62 (t, -$C\mathit{H}_2CH_2$-$N(CH_3)_2$, 6H), 2.35–2.39 (t, -$CH_2C\mathit{H}_2$-$N(CH_3)_2$, 6H), 2.22 (s, -$N(C\mathit{H}_3)_2$, 18H).

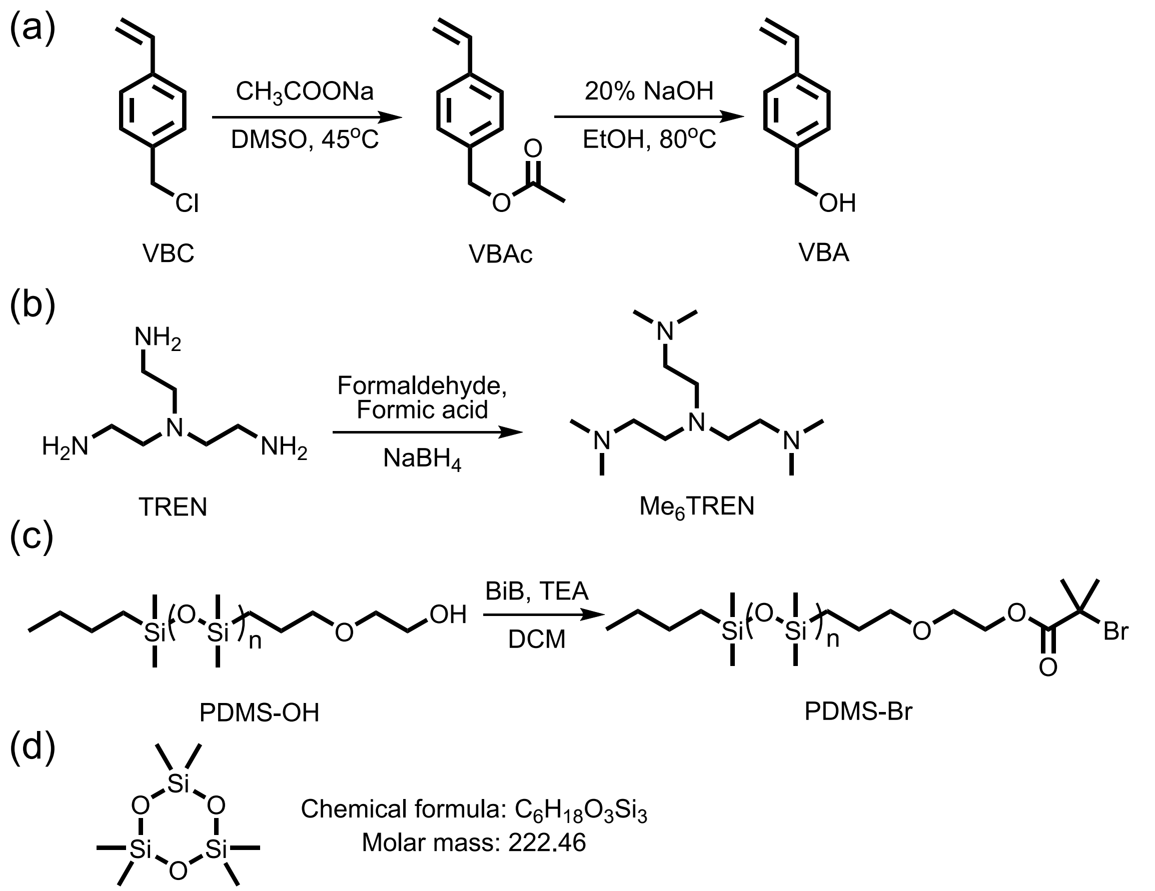

Scheme S1. Reaction steps for the syntheses of (a) VBA, (b) Me_6TREN, and (c) PDMS-Br (BiB: 2-bromoisobutyryl bromide). (d) Cyclic monomer of hexamethyl trisiloxane.

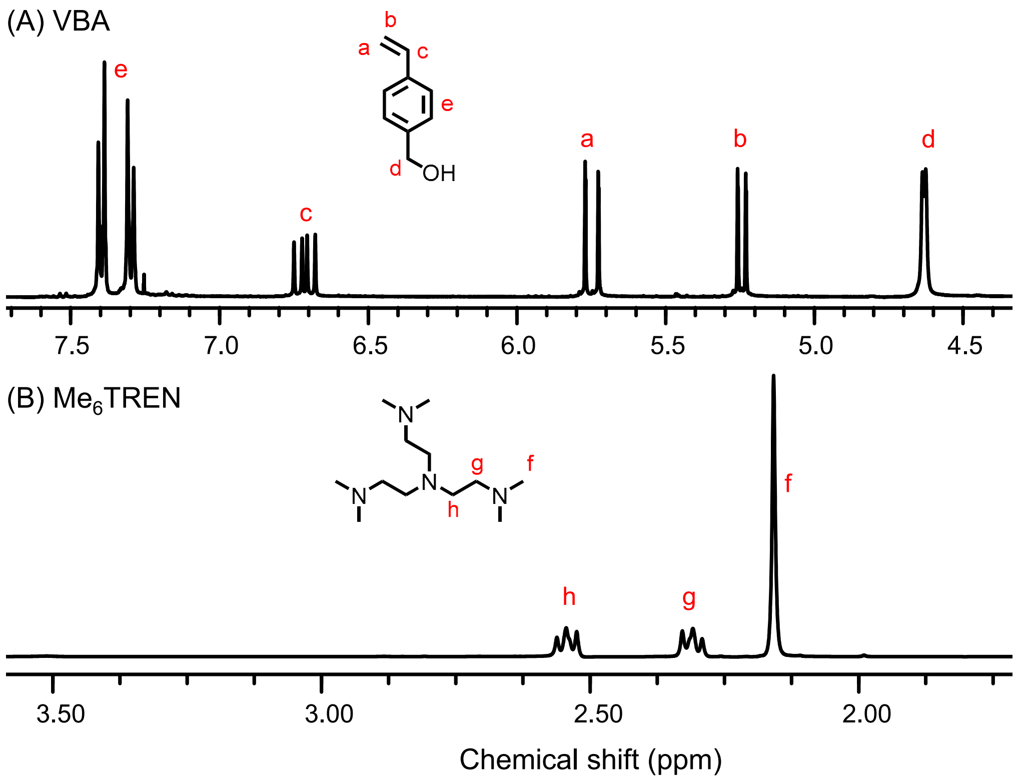

Figure S1. ^{1}H NMR spectra (400 MHz, $CDCl_3$) of (a) VBA and (b) Me_6TREN.

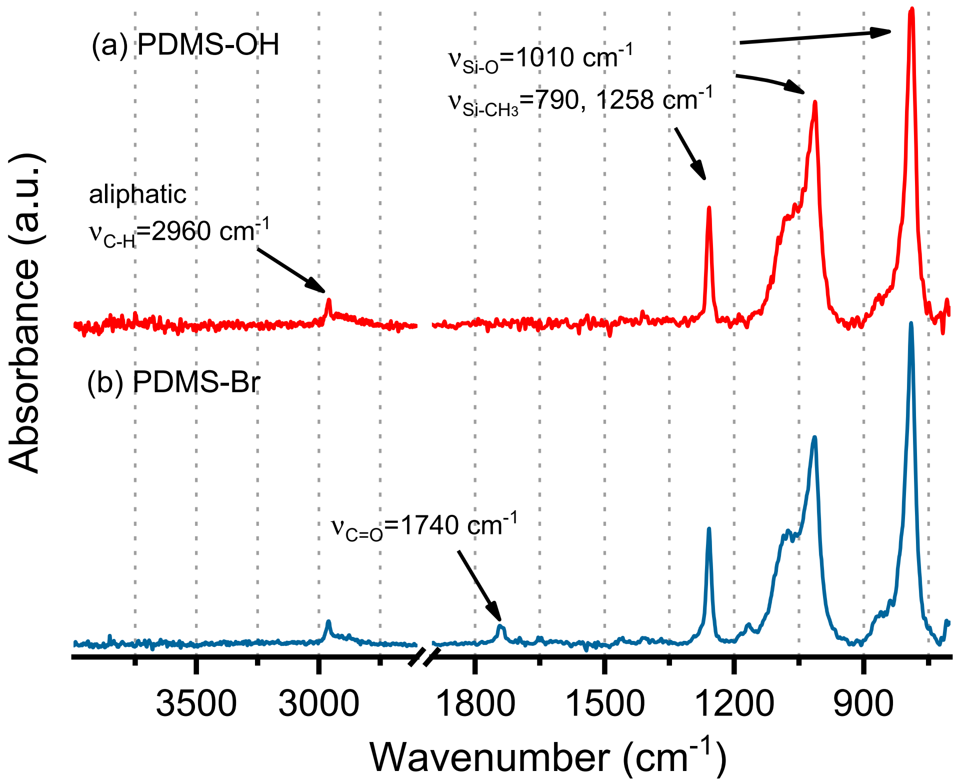

Figure S2. FT-IR spectra of (a) PDMS-OH and (b) PDMS-Br.

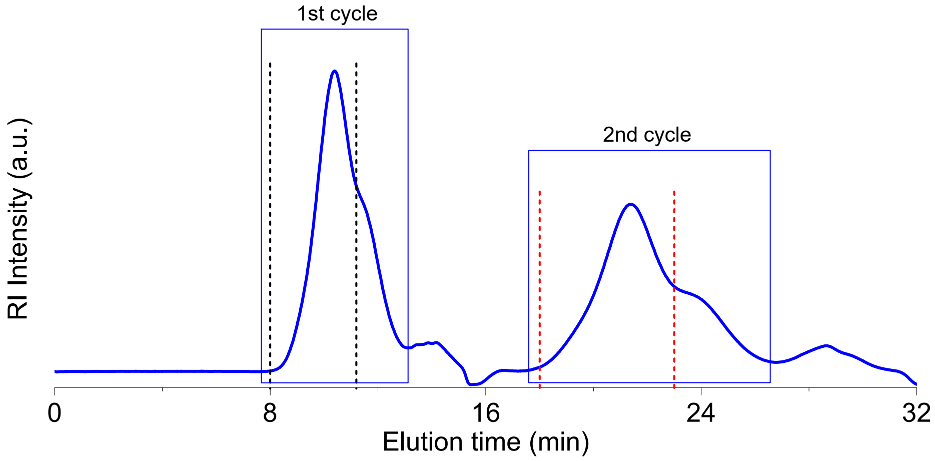

Figure S3. A consecutive rGPC trace for purifying a PDMS-μ-PCL crude product [red region: collections of well-defined μ-SCPs (i.e., elution time = ca. 18–23 min)].

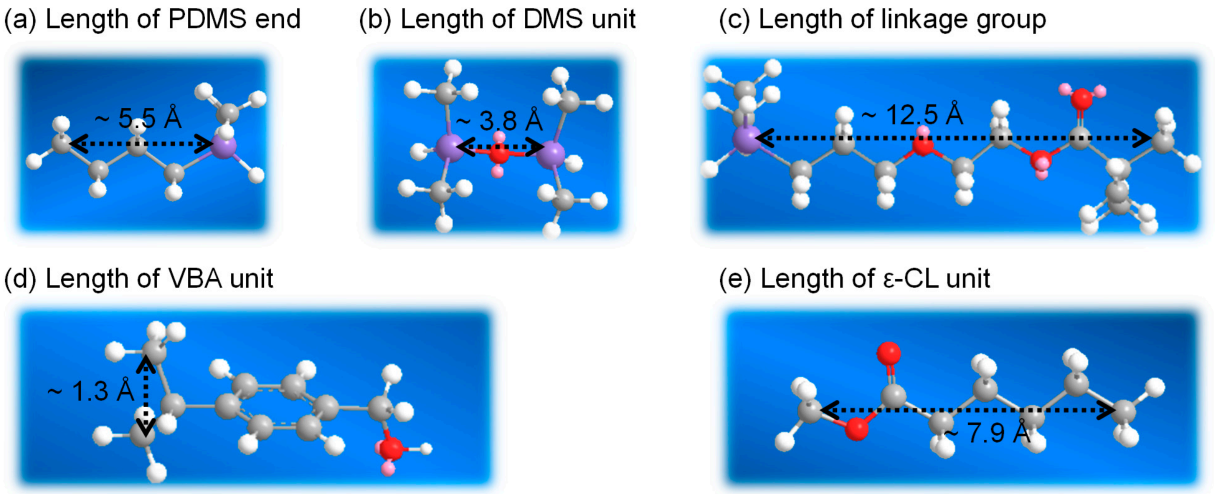

Figure S4. Spatial contour lengths of the relevant functionalities and monomer (estimated through MM2 molecular modeling) for $L_{\mu\text{-SCP1}}$ = 22.7 and $L_{\mu\text{-SCP2}}$ = 36.9 nm [estimation of the extended distance (L) using a set of PDMS end, $PDMS_n$ segment, PCL_p segment, linkage, and VBA unit].
